# Degradation of Bisphenol A by Nitrogen-Rich ZIF-8-Derived Carbon Materials-Activated Peroxymonosulfate

**DOI:** 10.3390/toxics12050359

**Published:** 2024-05-12

**Authors:** Xiaofeng Tang, Hanqing Xue, Jiawen Li, Shengnan Wang, Jie Yu, Tao Zeng

**Affiliations:** 1 Department of Environment Engineering, China Jiliang University, Hangzhou 310018, China; 2 Key Laboratory of Microbial Technology for Industrial Pollution Control of Zhejiang Province, Department of Environment, Zhejiang University of Technology, Hangzhou 310032, China; 3 Shaoxing Research Institute, Zhejiang University of Technology, Shaoxing 312000, China

**Keywords:** advanced oxidation processes, peroxymonosulfate activation, cage structure, metal sites, bisphenol A

## Abstract

Bisphenol A (BPA), representing a class of organic pollutants, finds extensive applications in the pharmaceutical industry. However, its widespread use poses a significant hazard to both ecosystem integrity and human health. Advanced oxidation processes (AOPs) based on peroxymonosulfate (PMS) via heterogeneous catalysts are frequently proposed for treating persistent pollutants. In this study, the degradation performance of BPA in an oxidation system of PMS activated by transition metal sites anchored nitrogen-doped carbonaceous substrate (M-N-C) materials was investigated. As heterogeneous catalysts targeting the activation of peroxymonosulfate (PMS), M-N-C materials emerge as promising contenders poised to overcome the limitations encountered with traditional carbon materials, which often exhibit insufficient activity in the PMS activation process. Nevertheless, the amalgamation of metal sites during the synthesis process presents a formidable challenge to the structural design of M-N-C. Herein, employing ZIF-8 as the precursor of carbonaceous support, metal ions can readily penetrate the cage structure of the substrate, and the N-rich linkers serve as effective ligands for anchoring metal cations, thereby overcoming the awkward limitation. The research results of this study indicate BPA in water matrix can be effectively removed in the M-N-C/PMS system, in which the obtained nitrogen-rich ZIF-8-derived Cu-N-C presented excellent activity and stability on the PMS activation, as well as the outstanding resistance towards the variation of environmental factors. Moreover, the biological toxicity of BPA and its degradation intermediates were investigated via the Toxicity Estimation Software Tool (T.E.S.T.) based on the ECOSAR system.

## 1. Introduction

In the context of advancing science and technology, the pace of industrial development continues unabated. Environmental pollution stemming from industrial activities poses an increasingly intricate challenge, given the complexity and stability of pollutant structures [1,2,3]. As a representative organic compound, bisphenol A (BPA) finds extensive application in the plastic industry, serving as a key monomer for the synthesis of epoxy resins and polycarbonates. Unfortunately, due to fluctuations in temperature and environmental pH, BPA tends to leach into the environment, potentially entering the human body via inhalation, ingestion, or dermal exposure. Moreover, as an estrogenic compound, BPA has the potential to affect estrogen receptor function, and it could induce endocrine-related cancers as it is absorbed by the human body through various pathways [4]. Peroxymonosulfate (PMS)-based advanced oxidation processes (AOPs) have emerged as a research hotspot in tackling sewage-related issues due to their simple operation and durability over diverse conditions in water matrices [5,6]. Unlike homogeneous transition metal ions-catalyzed PMS activation, which typically encounters the challenge of recyclability, heterogeneous activators hold great promise for facile recovery and regeneration [7]. Carbonaceous materials have been widely investigated for PMS [8], while their overly mild activation performance is the major issue impeding their potential application. Transition metal sites anchored nitrogen-doped carbonaceous substrate (M-N-C) materials were prospective candidates for overcoming the limitation, while the severe agglomeration of metal sites caused a troublesome setback for the utilization of the active metal sites [9].

As a novel category of heterogeneous activators, transition metal-anchored nitrogen-doped carbonaceous substrates were commonly synthesized utilizing zeolitic imidazolate frameworks (ZIFs) within metal–organic frameworks (MOFs) as templates [10,11,12]. As the ZIFs employed as the precursors for M-N-C materials, the N-linkers on the ordered structure of ZIFs serve as ligands for anchoring metal sites, thereby forming a cage structure that inhibits the agglomeration of metal atoms during the pyrolysis process. As a representative member of ZIFs, ZIF-8 material exhibits a crystalline phase with an ordered structure (typically in the range of 200–300 nm in particle size), rich porous structure, and high surface area [13,14]. Notably, the nitrogen-doped carbon material obtained from it typically exhibits a high nitrogen content, which facilitates the formation of a metal ions-anchored cage structure, thereby providing more accessible active centers. Copper doping facilitated the formation of Cu-N_4_ structures, leveraging the synergistic effects between copper and nitrogen atoms, thereby enhancing the catalytic efficiency. Subsequently, under inert gas protection, a judicious distribution of nitrogen atoms, copper atoms, and carbon atoms was achieved through high-temperature calcination, promoting the formation and exposure of active sites.

Nevertheless, M-N-C materials are still confronted with challenges related to low porosity and specific surface area, leading to a suboptimal exposure rate of active sites [15,16]. These limitations constrain the catalytic efficacy of M-N-C in redox reactions. Single-atom catalysts are acknowledged as possessing the smallest active site exposure among catalyst types and exhibit the highest atomic utilization rate [17,18,19]. However, single-atom catalysts are susceptible to atom migration and polymerization. Consequently, achieving the delicate balance between these factors necessitates a comprehensive understanding of materials, an exploration of the structure–activity relationship across various structures, and the design of catalyst materials with superior application and effectiveness, representing a pursuit of significant scientific importance. For transition metal-doped carbon and nitrogen materials, the approaches are typically categorized into two methods: in situ doping and post-treatment. In situ doping is a direct method that actively participates in the synthesis process during the formation of porous carbon nanostructures. Conversely, post-treatment involves subsequent steps to modify and treat the carbon material.

Herein, we utilized a straightforward two-step methodology, encompassing hydrothermal synthesis and high-temperature calcination, to prepare Cu-N-C, which was subsequently employed in the PMS activation process targeting the degradation of organic pollutants. ZIF-8 was employed as a precursor, leveraging the abundant N-linker nature to form the cage structure anchoring the metal centers. In this study, the feasibility of Cu-N-C/PMS systems for the degradation of BPA was investigated. The investigation delved into the impacts of diverse influential parameters (e.g., Cu-N-C content, PMS concentration, pH) on the removal of BPA. Additionally, an inquiry was made into the conceivable degradation mechanism and delineation of the potential pathway of BPA. Furthermore, the biological toxicity of both BPA and its degradation intermediates underwent assessment, employing the Toxicity Estimation Software Tool (T.E.S.T.) grounded on the ECOSAR system. The exploration of Cu-N-C offers unique insights into the application prospect of PMS-based AOPs and provides a new paradigm for utilizing Cu-N-C for the mineralization of pollutants.

## 2. Materials and Methods

### 2.1. Synthesis of N-C

Typically, 1.3 g 2-methylimidazole and 1.2 g Zn(NO_3_)_2_•6(H_2_O) were mixed in 30 mL methanol and subjected to ultrasonication for 15 min to obtain a homogeneous mixture. The mixture was added into a Teflon-lined autoclave (100 mL), followed by the hydrothermal reaction in an oven with a reaction temperature of 120 °C for 10 h, and then cooled to room temperature naturally to obtain precursor ZIF-8. After centrifugation, the product was placed in a quartz boat in a tube furnace. The temperature was ramped up to 900 °C with a heating rate of 5 °C min^−1^ and held for 3 h under an Ar atmosphere. Subsequently, the solid product was allowed to cool naturally to room temperature, followed by washing with deionized water and drying under vacuum at 60 °C for 12 h.

### 2.2. Synthesis of Cu-ZIF-8

In a typical synthesis, 1.3 g 2-methylimidazole was mixed into 15 mL methanol under vigorous magnetic stirring for 30 min to obtain solution A; 1.2 g Zn(NO_3_)_2_•6(H_2_O) and 26 mg copper(II) acetylacetonate (Cu(acac)_2_) were also mixed in 30 mL methanol and subjected to ultrasonication for 15 min to obtain solution B. Subsequently, solutions A and B were mixed under vigorous magnetic stirring for 60 min to obtain a homogeneous solution. The mixture was added into a Teflon-lined autoclave (100 mL) for hydrothermal reaction, which was conducted in an oven at 120 °C for 10 h. Subsequently, the reaction mixture was allowed to cool to room temperature naturally. The resulting precipitate was obtained, and impurities from free reagents were removed by centrifugation (15 min at 8000 rpm, repeated 3 times).

### 2.3. Synthesis of Cu-N-C

Typically, the synthesized Cu-ZIF-8 was placed in a quartz boat in a tube furnace. The temperature was increased to 900 °C with a heating rate of 5 °C min^−1^ and maintained for 3 h under an argon atmosphere. After cooling naturally to room temperature, the solid product was washed with deionized water and dried under vacuum at 60 °C for 12 h.

## 3. Results and Discussion

### 3.1. Material Characterization

**Figure 1:**
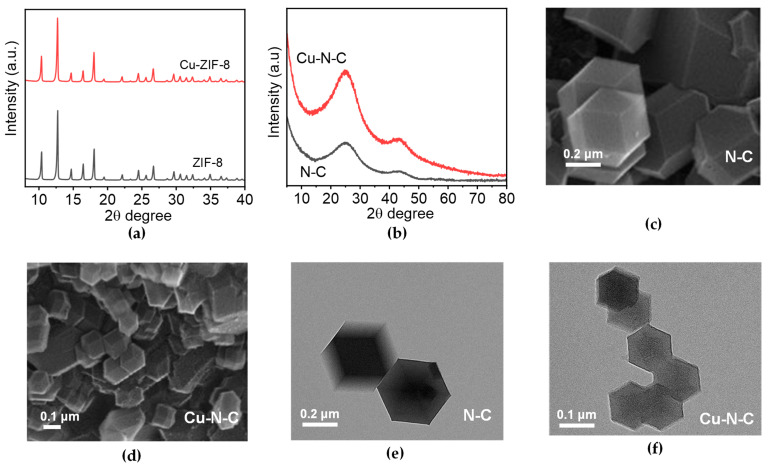
(**a**) XRD patterns of ZIF-8 and Cu-ZIF-8. (**b**) XRD patterns of N-C and Cu-N-C. SEM images of (**c**) N-C and (**d**) Cu-N-C. TEM images of (**e**) N-C and (**f**) Cu-N-C.

The components of the synthesized products were initially analyzed through powder X-ray diffraction (XRD). The sharp XRD peaks at 10.4°, 12.7°, 14.7°, 16.4°, and 18° correspond to the crystal planes of (002), (112), (022), (013), and (222) diffraction of ZIF-8, indicating high crystallinity of the samples (Figure 1a) [20,21]. In addition, Cu-ZIF-8 exhibits characteristic peaks located at the same positions as those of ZIF-8 (Figure 1a), indicating that the doping of transition metals (Cu) has a minimal impact on the structural integrity of ZIF-8, suggesting that the incorporation of transition metals can effectively preserve the properties of the template material, including high specific surface area, tunable porous structure, and excellent thermal and chemical resistance. However, the aforementioned characteristic peaks disappear in the XRD patterns of N-C and Cu-N-C after high-temperature calcination treatment. Instead, two characteristic diffraction peaks at 26° and 44° emerge, corresponding to the (002) and (100) graphite crystal planes, respectively (Figure 1b). Comparing the three carbon materials, it is observed that Cu-N-C has the most similar XRD patterns to the original template ZIF-8, which may be attributed to the fact that Cu^2+^ and Zn^2+^ share the same electron valence state, resulting in minimal influence on the zeolitic imidazole skeleton structure with Zn^2+^ as the core. However, due to the possibility of partial Cu^2+^ substitution for Zn^2+^, variations in peak area and half-width cannot be ignored, which may result in variations in particle size of these two materials. In summary, since the characteristic diffraction peaks share the same 2θ degree, the influence of transition metals on the skeleton structure during ZIF-8 formation, as indicated by XRD patterns, primarily manifests in material particle size, subsequently affecting surface area, pore size, and pore volume, while having no impact on the skeleton structure itself. Scanning electron microscopy (SEM) images of N-C and Cu-N-C shown in Figure 1c,d indicate that all the samples reveal a dodecahedral structure with uniform sizes and even distribution. The Cu-N-C catalyst particles exhibit a comparatively dense distribution, characterized by a particle size of approximately 120 nm. In contrast, the N-C catalyst particles demonstrate a more dispersed distribution, with a particle size around 260 nm. The transmission electron microscopy (TEM) images reveal the presence of surface wrinkles after high-temperature calcination (Figure 1e,f). Moreover, both samples exhibit a hollow interior, with a uniform distribution of metal on the material surface. This indicates that the overall skeleton structure of the material remains unaffected, further confirming the hypothesis of a cage-like structure. Honestly, since the main doping mechanism of Cu^2+^ involves entry into this cage structure, it cannot be denied that there is a minimal amount of Cu^2+^ substituting for Zn^2+^, forming a cage structure that facilitates catalytic reactions. As shown in Figure S1, elements C, N, O, Zn, and Cu are uniformly distributed on the surface of different sample materials, confirming that the high-temperature carbonization process must take place in an oxygen-free environment, which plays a crucial role in the uniform dispersion of transition metal atoms.

**Figure 2:**
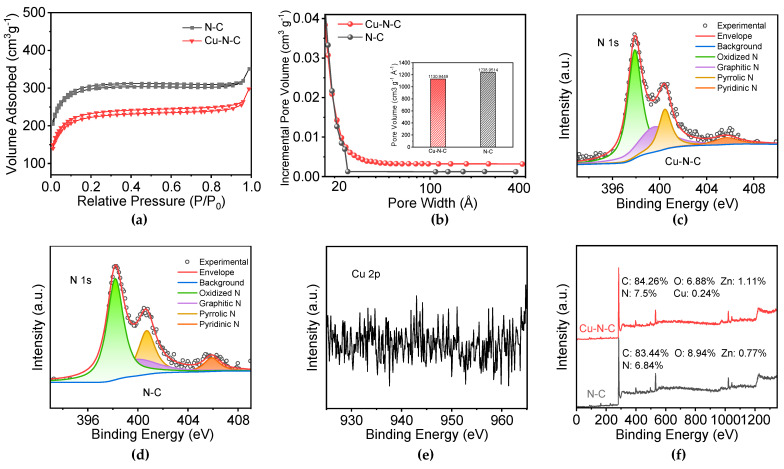
(**a**) N_2_ adsorption−desorption isotherms of N-C and Cu-N-C. (**b**) Pore size distribution curves for N-C and Cu-N-C. High−resolution XPS N 1s spectra of (**c**) N-C and (**d**) Cu-N-C. (**e**) XPS Cu 2p spectrum of Cu-N-C. (**f**) XPS survey spectrum and element content analysis of N-C and Cu-N-C.

Furthermore, Figure 2a presents the N_2_ adsorption–desorption isotherms, while the corresponding pore size distribution curves are depicted in Figure 2b for N-C and Cu-N-C. As for N-C and Cu-N-C, both the samples exhibited sharp inflections at a relative pressure of 0.0 < P/P_0_ < 0.1, which are characteristic of Type I isotherms commonly observed in microporous materials [22], providing evidence for the dominance of microporous structures in the pore network. The pore size distribution (Figure 2b) reveals that the doping of transition metals indeed affects the pore size distribution of the material. The incorporation of Cu elements shifts the original pore size distribution of the N-C sample towards mesopores (pore size ranging from 2 nm to 50 nm). Since the TEM elemental analysis indicates a low mass fraction of Cu atoms, suggesting minimal overall impact, the primary pore size remains dominated by micropores (pores with a size less than 2 nm). Moreover, regarding the specific surface area of the material, the doping of transition metals does have a certain impact. The doping of Cu elements has a relatively minor effect, causing a slight reduction in the specific surface area and pore volume of the Cu-N-C catalyst compared to the N-C sample material (from 1238.95 m^2^/g and 0.50 cm^3^/g to 1130.94 m^2^/g and 0.45 cm^3^/g, respectively).

To further investigate the chemical states and elemental composition of the samples, XPS measurements were performed. The N 1s XPS spectra (Figure 2c,d) for N-C and Cu-N-C could be deconvoluted into four peaks centered at 398.2, 399.8, 400.7, and 405.9 eV, corresponding to pyridine N, pyrrole N, graphitic N, and oxidized N, respectively [7,23]. The results reveal that nitrogen in Cu-N-C predominantly exists in the form of pyridinic nitrogen, constituting 50% of the total nitrogen content, accompanied by a simultaneous decrease in graphitic nitrogen. The Cu 2p XPS spectra (Figure 2e) indicate that the copper content in Cu-N-C is extremely low, making it challenging to detect, with minimal discernible signal and predominantly background intensity. However, the XPS elemental analysis results confirm the successful incorporation of Cu, but the content was extremely low, reaching only 0.24% (Figure 2f).

### 3.2. Oxyanions Activation Performance

**Figure 3:**
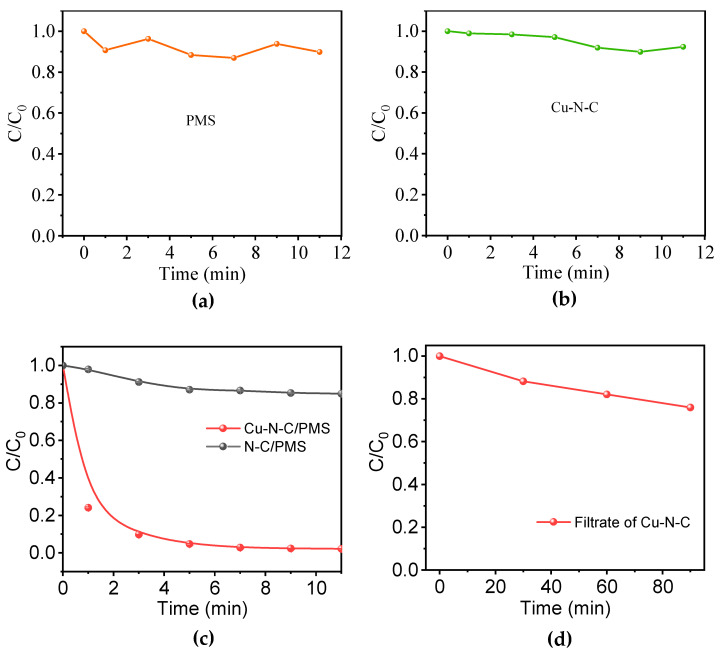
Elimination of BPA with the presence of (**a**) PMS and (**b**) Cu-N-C at different time intervals. (**c**) Elimination performance of BPA in different systems. (**d**) PMS activation performance of the filtrate of Cu-N-C. Routine condition: [catalyst] = 100 mg/L, [BPA] = 30 mg/L, [PMS] = 300 mg/L, temperature = 25 °C, and initial pH = 7.0.

The designed Cu-N-C catalyst particles aim to replace traditional homogeneous catalysts and noble metal catalysts to achieve the oxidation of BPA. The control experiments (Figure 3a,b) illuminate the limited contributions of PMS alone and the adsorption behavior of Cu-N-C to BPA removal. The limited oxidative impact of PMS on BPA could stem from the self-decomposition of PMS, which consequently yields sulfate radicals (SO_4_^•−^) that engage in the oxidation mechanism targeting BPA. As the N-C is fed into the system, the degradation efficiency significantly improved, reaching 48.25% within 90 min, indicating that nitrogen-doped carbon materials play a facilitating role in the activation of PMS (Figure 3c). The utilization of Cu-N-C as a catalyst for PMS activation results in the complete degradation of BPA within 11 min, which represents the state-of-the-art level among the polymeric materials and even rivals some single-atom catalysts in terms of performance (Table S1). This signifies a noteworthy advancement in degradation efficiency when compared to the utilization of N-C for PMS activation in the oxidation of BPA. The incorporation of transition metals such as Cu manifests a considerable enhancement in the catalytic activation of PMS, highlighting notable advantages in degradation efficiency.

It is well known that ions such as Cu, when subjected to high-temperature calcination, undergo a reduction process, transforming into atomic structures. The emergence of such atomic structures significantly reduces the demand for transition metal content, enabling efficient redox performance even at low doping concentrations. In the heterogeneous Cu-N-C/PMS systems, the reaction predominantly occurs at the interface between the solid and liquid phases, particularly within the framework cages. In this context, PMS comes into contact with copper atoms within the cages, becoming activated and generating positively charged copper ions. The generated copper ions interact with the outer layers of other negatively charged PMS anions, leading to minor electron rearrangements in PMS. This subtle rearrangement enhances the reactivity towards BPA. When the activated PMS comes into contact with organic molecules of BPA in the solution, it undergoes oxidation and decomposition.

In order to ascertain the contribution of copper ions freely released from the catalyst to the overall PMS activation performance, homogeneous catalysis was conducted using the filtrate from the catalyst in this experiment. To obtain the catalyst filtrate, the catalyst was added to deionized water in a ratio of 1 mg: 10 mL. The mixture was shaken in the dark for 90 min using a shaker. Subsequently, the catalyst was filtered out, and the resulting filtrate was collected. A 30 mg/L BPA aqueous solution was prepared using the filtrate, and an equivalent amount of PMS oxidant was introduced. Samples were collected at 30-minute intervals for subsequent analysis. From Figure 3d, it is evident that the filtrate of Cu-N-C does not exhibit any notably significant utility in catalyzing the PMS oxidation of BPA, indicating that the catalytic effect in the entire oxidation system is not attributed to the catalytic activity of freely released transition metal ions such as copper. The data obtained from the atomic absorption spectrophotometer in Table 1 reveals that after 90 min of shaker agitation, no metal ions have separated from the catalyst. This further underscores that the catalytic activity in the entire oxidation system is attributed to the Cu-N-C catalysts.

### 3.3. Catalytic Performance of Catalysts Prepared with Different Metal Precursors

**Figure 4:**
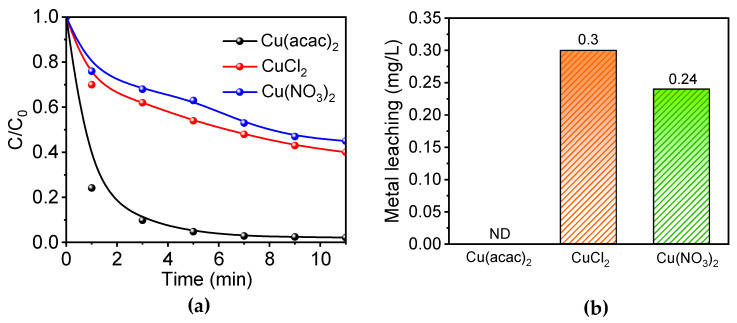
(**a**) PMS activation performance of catalysts prepared from different copper precursors. (**b**) Metal leaching of Cu-N-C catalysts prepared by different precursors. Routine condition: [catalyst] = 100 mg/L, [BPA] = 30 mg/L, [PMS] = 300 mg/L, temperature = 25 °C, and initial pH = 7.0.

From Figure 4a, it is evident that the catalytic activity varies significantly depending on the transition metal precursor (Cu(acac)_2_, CuCl_2_, and Cu(NO_3_)_2_) used in the preparation of the catalyst. As ZIF-8 serves as the framework structure, the ions in the transition metal precursor are enveloped by the cage structure of ZIF-8. Since there is a considerable difference in molecular diameters, copper acetylacetonate molecules, being larger than copper chloride and copper nitrate molecules but slightly smaller than the diameter of the cage structure, are typically encapsulated one at a time within a single cage structure. In contrast, for copper chloride and copper nitrate, multiple molecules are often encapsulated, making it prone to transition metal accumulation and metal aggregation phenomena. The degradation experiment results demonstrate that catalysts prepared with copper acetylacetonate as the copper precursor exhibit superior catalytic activity. This observation indirectly supports the notion that employing copper acetylacetonate as the Cu source favors the formation of a higher number of catalytically active sites. Building upon the earlier discussion, the formation of CuN_4_ is more favorable when one copper acetylacetonate corresponds to one cage structure. In contrast, multiple copper chloride or copper nitrate molecules corresponding to one cage structure are more likely to be connected to carbon, masking the CuN_4_ structure and consequently reducing the overall catalytic activity.

In order to investigate the structural stability of catalysts prepared with different copper-containing precursors (Cu(acac)_2_, CuCl_2_, Cu(NO_3_)_2_), we conducted metal leaching tests on the catalysts after the reaction. As depicted in Figure 4b, Cu-N-C catalysts synthesized from CuCl_2_ and Cu(NO_3_)_2_ precursors demonstrated average leaching values of 0.3 mg and 0.24 mg, respectively. Conversely, Cu leaching was imperceptible in the three experiments utilizing Cu(acac)_2_ as the precursor, indicating that Cu-N-C prepared with Cu(acac)_2_ as the precursor possesses structural stability advantages.

### 3.4. Metal Doping Concentration-Dependent Catalytic Activity of Cu-N-C

**Figure 5:**
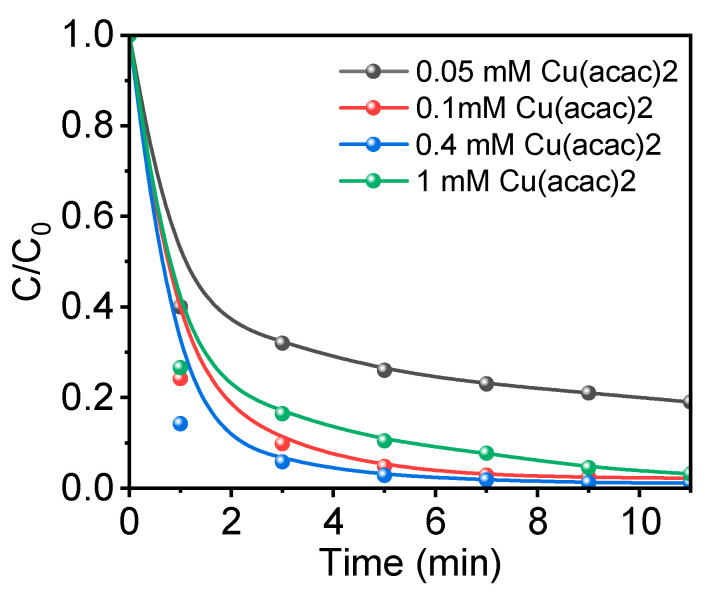
BPA degradation performance of Cu-N-C synthesized by Cu(acac)_2_ in different concentrations. Routine condition: [catalyst] = 100 mg/L, [BPA] = 30 mg/L, [PMS] = 300 mg/L, temperature = 25 °C, and initial pH = 7.0.

The experimental results depicted in Figure 5 highlight the impact of varying concentrations of copper acetylacetonate as a precursor upon the catalytic activity of Cu-N-C catalysts. Accordingly, with a lower concentration of Cu(acac)_2_ (0.05–0.4 mM), the catalytic activity of the Cu-N-C catalyst improves as the precursor concentration increases. Nonetheless, a decrease in catalytic performance is observed when the concentration reaches 1 mM. This observation reinforces the notion that excessive doping levels may inhibit the original catalytic activity of the catalyst, potentially ascribing to the formation of inactive catalytic sites. While the molecular size of copper Cu(acac)_2_ is relatively large, allowing for the encapsulation of at most one molecule in a single cage structure, an excess of transition metal can disrupt the ZIF-8 framework, reducing pore volume, specific surface area, and potentially causing local structural collapses. In line with the aforementioned characterization results, copper doping does result in a slight decrease in material-specific surface area and pore volume. Nevertheless, Cu-N-C catalysts inherit the thermal stability from the ZIF-8 material, and they acquire the catalytic and magnetic properties introduced by Cu^2+^, resulting in an overall enhancement of catalytic performance.

Moreover, the effective utilization of copper elements varies with different concentrations of the metal precursor. As depicted in Figure S2, the doping ratio is highest when the precursor concentration is 0.1 mM, indicating optimal utilization of Cu(acac)_2_. This not only ensures high catalytic activity but also enhances the efficiency of raw material utilization, leading to cost savings in large-scale catalyst production. Additionally, as depicted in Figure S3, when the doping level of Cu(acac)_2_ reaches 0.4 mM, there is evidence of copper element precipitation. Therefore, based on the preceding conclusions, a concentration of 0.1 mM for Cu(acac)_2_ is selected.

### 3.5. Effect of Catalyst and PMS Dosage

**Figure 6:**
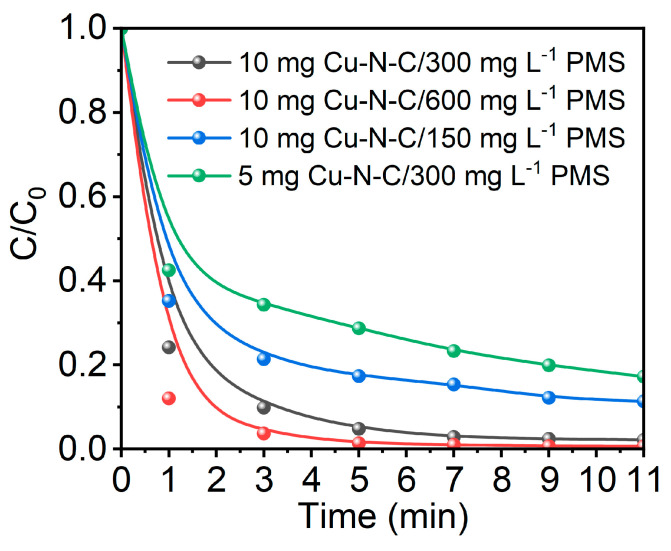
BPA degradation performance of the Cu-N-C/PMS system under different conditions. Routine condition: [BPA] = 30 mg/L, temperature = 25 °C, and initial pH = 7.0.

Upon elevating the concentration of the oxidant PMS from 150 mg/L to 300 mg/L, a notable acceleration in the overall degradation process is observed, and the ~99% BPA removal rate could be achieved within 5 min (Figure 6). A further increase in the PMS dosage led to only a minor improvement in the overall BPA removal efficiency. This could be attributed to the limited availability of reactive sites on the fixed amount of Cu-N-C catalyst for the excess PMS molecules. Furthermore, reducing the catalyst dosage from 10 mg to 5 mg significantly decelerates the catalytic activation of PMS for BPA oxidation, highlighting the substantial impact of catalyst dosage on degradation performance. The combined analysis of the impact of changes in PMS and catalyst dosages on the degradation efficiency reveals that an increase in PMS dosage results in a higher contact tendency between PMS ions and the active sites of the catalyst, accelerating the production of SO_4_^•−^. Cu-N-C, relying on ZIF-8 as a template, possesses a large surface area, facilitating the transfer of PMS ions and SO_4_^•−^ with reduced congestion during internal catalyst transport, leading to a decrease in the reaction rate. The large pore volume and porous structure expedite the accumulation and transport of PMS ions and SO_4_^•−^, facilitating both PMS activation and the transport of SO_4_^•−^. This, in turn, enhances contact with BPA molecules, expediting the overall degradation process. Conversely, a decrease in oxidant quantity reduces the influx of PMS into the Cu-N-C catalyst, potentially leaving some active sites idle and slowing down the overall oxidative degradation process. Simultaneously, changes in catalyst quantity fundamentally reduce the number of active sites, resulting in a decrease in the overall reaction rate. In conclusion, the optimal PMS dosage is not necessarily the highest, as there exists a limit to its impact on reaction efficiency, and the excessive catalyst dosage may lead to wastage, incurring additional costs. Therefore, the appropriate ratio of PMS, catalyst, and organic pollutant concentrations becomes crucial. The selection of these proportions in this experiment takes into account a comprehensive evaluation of factors such as cost and reaction rate.

### 3.6. Effect of pH Value

**Figure 7:**
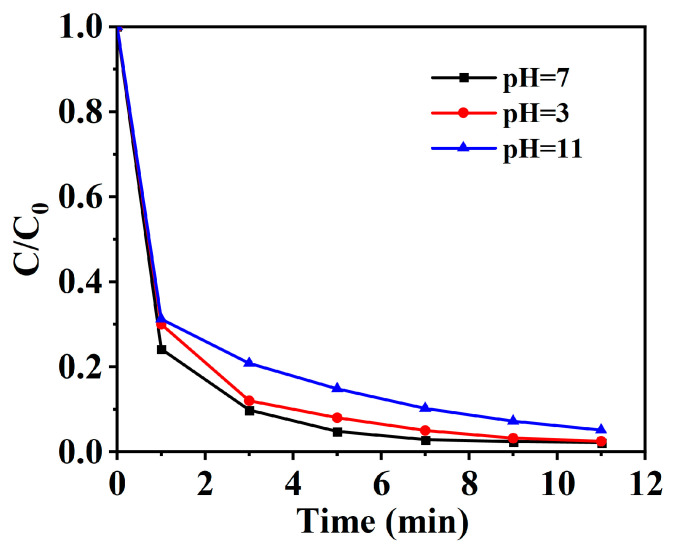
BPA degradation performance of the Cu-N-C/PMS system under different pH values. Routine condition: [catalyst] = 100 mg/L, [BPA] = 30 mg/L, [PMS] = 300 mg/L, temperature = 25 °C.

In the process of pollutant degradation, the actual treatment of water bodies is often complicated and unstable. Therefore, the actual treatment needs to consider the change in water environment factors such as water pH value. To enhance the catalytic applicability across different pH conditions, simulated wastewater solutions of BPA were prepared at initial pH values of 3, 7, and 11 by adjusting with HCl and NaOH solutions. As depicted in Figure 7, the Cu-N-C catalyst maintains high catalytic activity over a wide range of pH conditions, achieving complete degradation of BPA within 15 min. This underscores the broad pH applicability of the Cu-N-C/PMS system. Although a marginal reduction in catalytic activity is noted under acidic and alkaline conditions in comparison to neutral conditions, this phenomenon is likely ascribed to the influence of hydrogen ions and hydroxide ions on PMS activation. Nevertheless, the BPA degradation efficiency in the Cu-N-C/PMS system remains consistently high under both acidic and basic conditions, demonstrating its inherent resistance to pH variations. Therefore, based on the experimental findings, BPA degradation within the Cu-N-C/PMS system exhibits sustained efficacy across a range of pH environments.

### 3.7. Degradation Performance of Cu-N-C/PMS to Other Representative Organic Contaminants

In the current industrial development process, various chemical reagents are employed across diverse production processes. The intricate nature of these processes results in wastewater characterized by a multitude of chemical substances with diverse structures. Consequently, the treatment of such wastewater often necessitates an oxidation system capable of addressing a broad spectrum of organic pollutants, imposing stringent requirements on the catalytic performance of the catalyst. The central focus of this research is to investigate and develop a catalyst that can exhibit excellent catalytic activation performance for wastewater treatment in various application domains.

**Figure 8:**
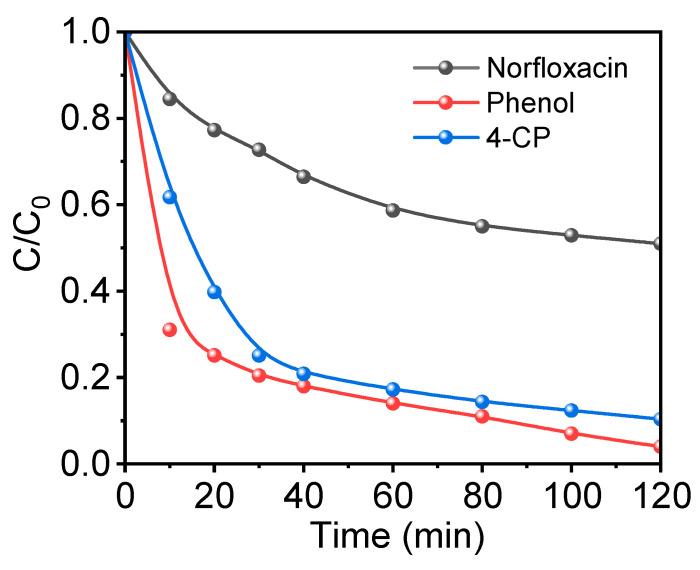
Degradation of various organic contaminants in the Cu-N-C/PMS system. Routine condition: [catalyst] = 100 mg/L, [contaminant] = 30 mg/L, [PMS] = 300 mg/L, temperature = 25 °C, and initial pH = 7.0.

In the realm of pharmaceutical production and various industrial processes, phenol stands as a commonly employed reagent or an intermediate byproduct. Its inherent benzene ring structure imparts a degree of acidity to its presence in aqueous solutions. Despite its structural simplicity compared to other organic pollutants, the significance of phenol in phenolic wastewater arises from its sheer volume and elevated concentration. Consequently, the treatment of phenolic wastewater demands highly efficient catalytic oxidation systems. The results depicted in Figure 8 showcase that the Cu-N-C/PMS system attains a substantial 70% degradation of phenol within the initial 10 min of the reaction, ultimately achieving complete degradation by 120 min. This underscores the remarkable phenol degradation capabilities exhibited by the Cu-N-C/PMS system.

In comparison to phenol, the organic pollutant 4-chlorophenol (4-CP) introduces halogens in addition to the hydroxyl group on the benzene ring. The inclusion of halogens elevates the demands on catalytic oxidation systems. This is attributed to the need for initial dehalogenation in the degradation process of organic pollutants containing halogens, followed by the mineralization of the organic compound to achieve complete decomposition. As depicted in Figure 8, the degradation kinetics of 4-CP within the Cu-N-C/PMS system exhibit a relatively sluggish pace compared to phenol, primarily attributed to the supplementary dehalogenation process integral to the complete decomposition of 4-CP. Within 120 min, a degradation exceeding 90% of 4-chlorophenol is attained, underscoring the efficacy of the Cu-N-C/PMS system in the degradation of halogenated organic pollutants, thus broadening its applicability spectrum.

Simultaneously, norfloxacin was employed as a target contaminant to investigate the elimination efficiency of the Cu-N-C/PMS system toward complex and polyaromatic compounds containing fluorine halogens. As illustrated in Figure 8, the degradation rate of norfloxacin is notably slower in comparison to phenol and 4-CP. Specifically, the Cu-N-C/PMS system exhibits a degradation exceeding 40% of norfloxacin within 120 min, suggesting its efficacy in eliminating halogenated complex organic compounds.

### 3.8. Recyclability of Cu-N-C

**Figure 9:**
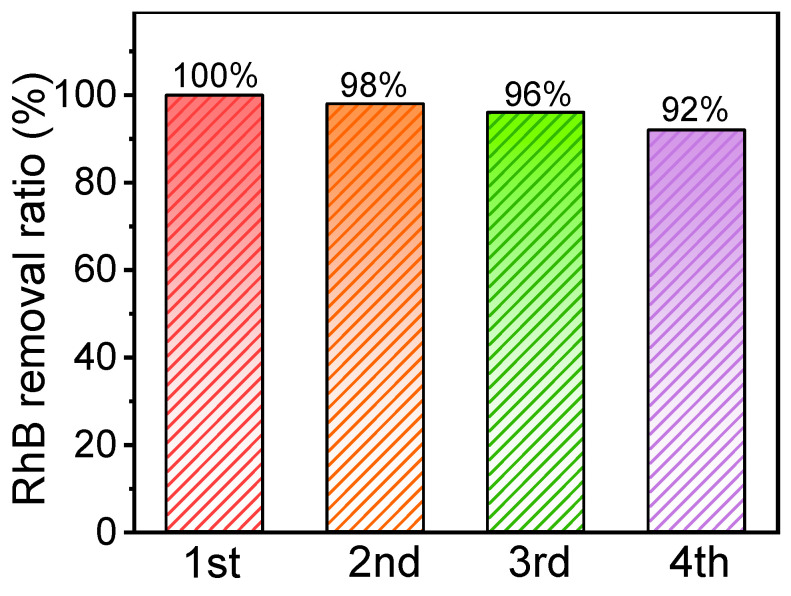
Recyclability of the Cu-N-C/PMS system for BPA removal. Routine condition: [catalyst] = 100 mg/L, [BPA] = 50 mg/L, [PMS] = 30 mg/L, temperature = 25 °C, and initial pH = 7.0.

The stability and recyclability of the materials were deemed essential factors for heterogeneous reactions. To assess the stability and recyclability of Cu-N-C, the used Cu-N-C was separated and collected by suction filtration, followed by repeated washing with methanol and deionized water. As depicted in Figure 9 and Figure S4, the Cu-N-C/PMS system consistently achieved removal rates of 100%, 98%, 96%, and 92% for BPA across the four runs. The slight reduction in degradation efficiency may be attributed to a marginal catalyst powder loss. Nonetheless, the overall degradation rate of BPA remained above 90%, underscoring the favorable recyclability of Cu-N-C.

### 3.9. TOC Removal Performance

**Figure 10:**
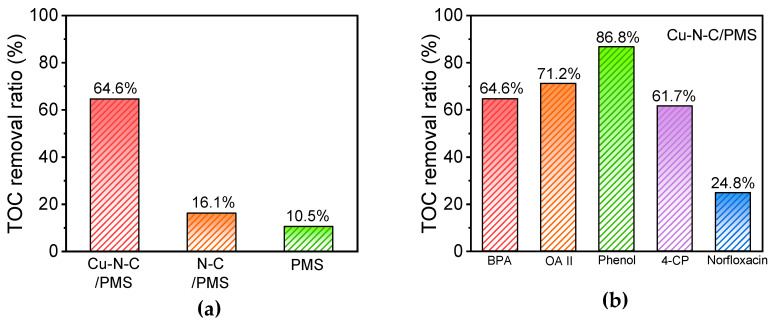
(**a**) The mineralization of BPA in different systems. (**b**) The mineralization of various organic contaminants in the Cu-N-C/PMS system. Routine condition: [catalyst] = 100 mg/L, [contaminant] = 50 mg/L, [PMS] = 300 mg/L, temperature = 25 °C, reaction time = 60 min.

The mineralization of BPA encompasses a complex process wherein BPA molecules undergo decomposition into smaller molecular intermediates, thereby effectively diminishing the environmental toxicity associated with the original BPA compound. Thus, the rate of mineralization of organic contaminants stands as a crucial benchmark for assessing the efficacy of an oxidation system in degradation processes [24]. To determine the mineralization rate of BPA in different systems, we utilized a TOC-L analyzer (Shimadzu TOC-L, Kyoto, Japan) to analyze the TOC removal efficiency. As illustrated in Figure 10a, the Cu-N-C/PMS system demonstrated a BPA mineralization rate exceeding 60% within 60 min. In contrast, the TOC removal rate of BPA using the N-C sample remained at a mere 16%. This observation underscores the potential of transition metal doping to augment the overall catalytic activation performance of the N-C structure. Furthermore, we explored the catalytic efficacy of Cu-N-C across varying pH levels (Figure S5). The finding reveals that at pH values of 3, 7, and 11, the Cu-N-C/PMS system achieved mineralization rates of BPA at 64.6%, 61.2%, and 58.8%, respectively, underscoring its mineralization effectiveness across acidic, neutral, and alkaline environments.

In evaluating the efficacy against Norfloxacin as the target contaminant, the Cu-N-C/PMS system exhibited limited degradation efficiencies, achieving only 24.8% and 22.1%, respectively (Figure 10b). Conversely, when targeting pollutants such as BPA, Acid Orange II (AO II), phenol, and 4-CP, both systems displayed noteworthy total organic carbon (TOC) removal rates (>60%). This underscores the effective mineralization potential of both systems for relatively simpler organic contaminants.

## 4. Pathways of BPA Degradation by M-N-C-Activated PMS and Toxicity Analysis

**Figure 11:**
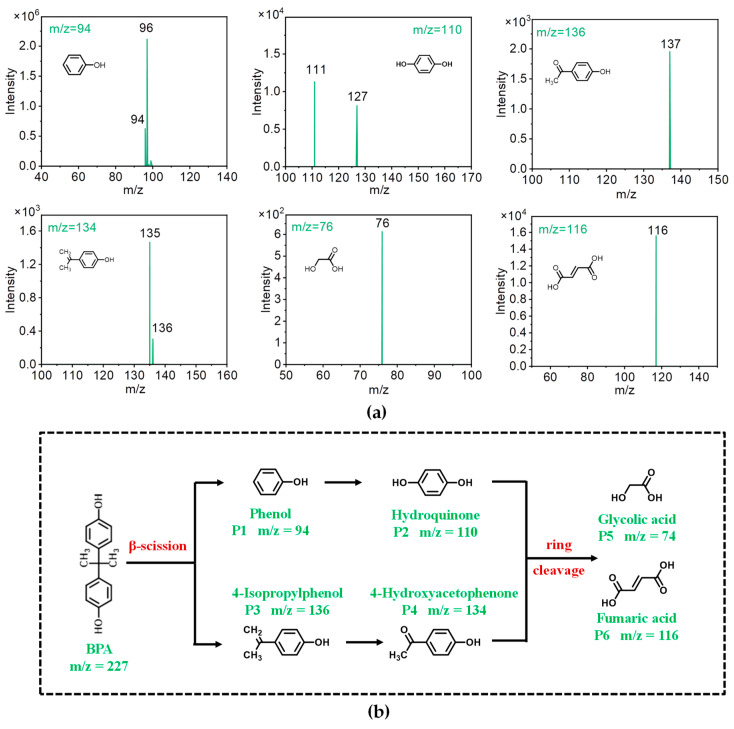
(**a**) Mass spectra of the detected BPA intermediates in the Cu-N-C/PMS system. (**b**) Presentation of the proposed degradation pathway of BPA in the PTEB-F_15_/PMS/vis system.

To elucidate the degradation process of BPA under the oxidative influence of M-N-C-activated PMS, we analyzed the intermediates of BPA degradation using an LC/MS system. An investigation into the degradation pathway and corresponding toxicity analysis was undertaken within the Cu-N-C/PMS system. Six degradation intermediates of BPA were detected and identified (Figure 11a), characterized by the mass-to-charge ratios (*m*/*z*) of 94, 110, 136, 134, 74, and 116, respectively. Therefore, the degradation pathway of BPA in the Cu-N-C/PMS system could be elucidated on the basis of analytical results of LC/MS. The pathway for the BPA degradation primarily included β-scission and ring cleavage (Figure 11b) [25,26,27,28,29]. Specifically, BPA underwent an initial β-scission, leading to the formation of phenol (P1) and 4-isopropylphenol (P3) with *m*/*z* of 94 and 136, respectively. Subsequently, the mono-hydroxylated intermediates P1 and P3 would further transform into hydroquinone (P2) and 4-hydroxyacetophenone (P4). These generated P2 and P4 were susceptible to the oxidation effect of Cu-N-C-activated PMS and underwent ring cleavage to generate simple organic acids, including glycolic acid (74) and fumaric acid (116), which ultimately mineralized into CO_2_ and H_2_O.

**Figure 12:**
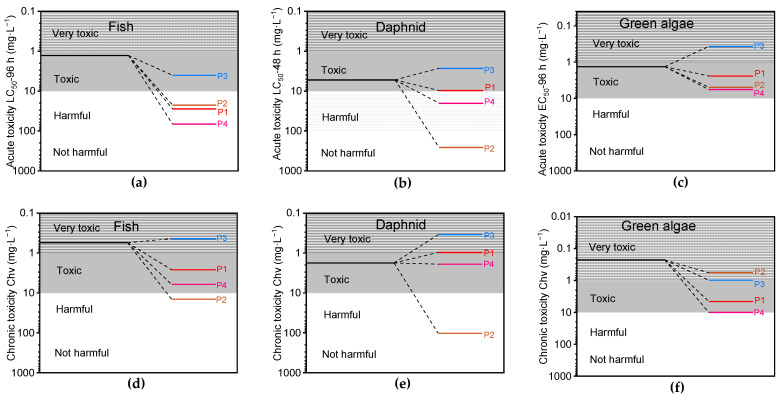
Acute toxicity LC_50_ of fish (**a**) and daphnid (**b**). Acute toxicity EC_50_ of green algae (**c**). Chronic toxicity Chv of fish (**d**), daphnid, (**e**) and green algae (**f**).

In addition, the corresponding acute and chronic toxicity of the generated intermediates were predicted using a Toxicity Estimation Software Tool (T.E.S.T.) based on the ECOSAR system [30,31,32]. Based on the Globally Harmonized System of Classification and Labelling of Chemicals, BPA and its degradation intermediates were classified into four toxicity levels (Figure 12 and Table 2). Based on the toxicity analysis findings, most of the β-scission products displayed significantly reduced toxicity compared to the initial BPA, with the exception of 4-isopropylphenol (P3), which demonstrated comparable toxicity to the original BPA. It is worth mentioning that the ring cleavage products P5 and P6 exhibited markedly reduced acute and chronic toxicities, as evidenced by their extremely high LC50, EC50, and ChV values, thus classifying them as “not harmful” products. This finding suggests a potential for further reduction in toxicity following β-scission product reactions. Moreover, the comprehensive examination of the aforementioned findings reveals a significant reduction in BPA toxicity subsequent to treatment in the M-N-C/PMS system.

## 5. Conclusions

This work presents a straightforward one-step synthesis of transition metal Cu-ZIF-8 material, followed by the preparation of Cu-N-C catalyst through high-temperature calcination. This study demonstrates that the transition metal atoms enclosed within this cage structure exhibit outstanding catalytic oxidation effects. Experimental results show the efficacy of Cu-N-C in activating PMS for the degradation of BPA. The Cu-N-C/PMS system exhibited superior BPA elimination efficiency over a wide pH range. More importantly, the Cu-N-C/PMS system demonstrates effective degradation potential towards a variety of organic pollutants, highlighting its excellent potential for practical applications. The synthesized Cu-N-C not only proves effective in degrading BPA but also demonstrates potential in the non-selective degradation of organic pollutants, achieving excellent mineralization effects. Additionally, the toxicity of original BPA and its decomposition intermediates was evaluated by the T.E.S.T. model, and most toxicity indicators suggested that the Cu-N-C/PMS system has the potential to offer a step-by-step de-toxicity process to relieve the final biological hazard. More importantly, the application concept of employing the Cu-N-C/PMS system provides a certain theoretical basis and fundamental support for tackling organic contaminant-related environmental issues.

## Figures and Tables

**Table 1:** The content of metal ions in filtrate.

Catalyst	Content of Metal Ion
Filtrate of Cu-N-C	ND

**Table 2:** Acute toxicity and chronic toxicity of products 5 and 6.

Possible Products	Acute Toxicity (mg·L^−1^)			Chronic Toxicity (mg·L^−1^)		
	Fish	Daphnid	Green Algae	Fish	Daphnid	Green Algae
	(LC_50_)	(LC_50_)	(EC_50_)	(ChV)	(ChV)	(ChV)
P5	53,300	25,300	8960	4220	1500	1570
P6	355,000	152,000	35,100	24,800	6730	4890

## Data Availability

Data are available from the corresponding authors upon request.

## Supplementary Materials

The following supporting information can be downloaded at: https://www.mdpi.com/article/10.3390/toxics12050359/s1, Figure S1: Elemental mapping images of (a) N-C and (b) Cu-N-C; Figure S2: The Cu doping content and ratio of the catalysts prepared with different transition metal precursor concentrations. Routine condition: [catalyst] = 100 mg/L, [BPA] = 30 mg/L, [PMS] = 300 mg/L, temperature = 25°C, and initial pH = 7.0, reaction time = 11 min; Figure S3. Metal leaching of Cu-N-C catalysts prepared by precursors in different concentration. Routine condition: [catalyst] = 100 mg/L, [BPA] = 30 mg/L, [PMS] = 300 mg/L, temperature = 25°C, and initial pH = 7.0, reaction time = 11 min; Figure S4. The chromatogram spectra of BPA in the four cycling experiments; Figure S5. The mineralization of the BPA in Cu-N-C/PMS system under different pH values. Routine condition: [catalyst] = 100 mg/L, [contaminant] = 50 mg/L, [PMS] = 300 mg/L, temperature = 25°C, reaction time = 60 min; Table S1: The comparison of catalytic performance for polymeric catalysts in PMS activation [33,34,35,36,37,38,39,40].
