# Supplementary material for: Degradation of Bisphenol A by Nitrogen-Rich ZIF-8-Derived Carbon Materials-Activated Peroxymonosulfate
Source: Toxics. 2024 May 12;12(5):359. doi: 10.3390/toxics12050359 (PMC11125605; doi:10.3390/toxics12050359)
Supplement: Supplementary file 1 [file toxics-12-00359-s001.zip › toxics-2948187-SI.pdf]

Supporting Information

Degradation of Bisphenol A by Nitrogen-Rich ZIF-8-Derived Carbon Materials-activated Peroxymonosulfate

Xiaofeng Tang [1, 2], Hanqing Xue [1], Jiawen Li [1], Shengnan Wang [2], Jie Yu [1, *] and Tao Zeng [2, 3]

1 Department of Environment Engineering, China Jiliang University, Hangzhou 310018, PR China

2 Key Laboratory of Microbial Technology for Industrial Pollution Control of Zhejiang Province, Department of Environment, Zhejiang University of Technology, Hangzhou, Zhejiang, 310032, P.R. China

3 Shaoxing Research Institute, Zhejiang University of Technology, Shaoxing, Zhejiang 312000, P.R. China

* Correspondence: yujie@cjlu.edu.cn

Text S1. Chemicals and Reagents.

Bisphenol A (BPA), Acid Orange (II) (AO(II)), phenol, 4-chlorophenol (4-CP) and norfloxacin were obtained from J&K Chemical Co., Ltd. Copper (II) acetylacetonate (Cu(acac)$_2$), Iron(Ⅲ) acetylacetonate (Fe(acac)$_3$), Potassium monopersulfate triple salt (PMS, Oxone®, $KHSO_5 \cdot 0.5KHSO_4 \cdot 0.5K_2SO_4$), were purchased from Sigma-Aldrich Chemical Co., Ltd. Zinc nitrate ($Zn(NO_3)_2$) and 2-methylimidazole were purchased from Aladdin Co., Ltd. Ethanol (EtOH) and methanol (MeOH) were obtained from Sinopharm Chemical Reagent Co. Ltd. (Shanghai, China). All chemicals used in this study were at least in analytical grade without any further purification. Ultrapure water from a Milli-Q water system was used through the experiments.

Text S2. Experimental Procedure.

The catalytic performance of Cu-N-C and Fe-N-C samples was evaluated in a PMS solution for BPA degradation. In a typical experiment, 10 mg catalyst was added into 100 mL solution containing 30 mg/L BPA and 30 mg of PMS in a conical flask. The reaction was initiated by shaker stirring at 250 rpm at 25 ℃ ± 1℃. At predetermined time intervals, the 1.0 mL of solution was acquired for analysis.

Text S3. Analytic Methods.

The concentration of BPA was analyzed by an HPLC system (Waters e2695), equipped with a UV detector and a C18 column (4.6 mm × 150 mm, 5

μm). The mobile phases and detection wavelengths were set as follows: methanol/water (70:30, v/v) with λ = 225, 210 and 269 nm for BPA, phenol and 4-CP, respectively.

Liquid chromatography/mass spectrometry (LC/MS) spectrometry were carried out using an Agilent 1290 (Agilent, USA) liquid chromatograph equipped with a QQQ (Agilent6460, USA) tandem mass spectrometer.

Crystal structure and morphology of the catalysts were examined by scanning electron microscopy (S−4800, Hitachi, SEM) and transmission electron microscopy (Tecnai G2 F30 S−Twin, Netherlands, TEM).

The specific surface area and pore size distributions of samples were analyzed by N_2 adsorption/desorption at −196 °C and calculated by BET method (ASAP−2010 Analyzer, Micromeritics).

Powder X−ray diffraction (XRD) patterns were conducted by SCINTAG X'TRA X−ray diffractometer (Thermo ARL) using Cu−Kα radiation operating at 45 kV and 40 mA in the 2θ range of 10–80°.

The contents of Fe in the samples were quantified by an inductively coupled plasma optical emission spectrometer (ICP−OES).

X−ray photoelectron spectroscopy (Thermo Scientific K−Alpha, XPS) was used to determine the chemical compositions of samples using Al−Kα radiation (1486.6 eV).

The degradation of BPA was fitted by the pseudo first − order kinetic simulation equation (1):

$$\ln\frac{C_t}{C_0}=-k_{obs}t \qquad (1)$$

where C_0 is the initial concentration of BPA (mg L^{-1}), C_t is the concentration of BPA in the specific time (t) during the reaction (mg L^{-1}), k_{obs} is the observed first−order rate constant (min^{-1}) and t is the reaction time (min).

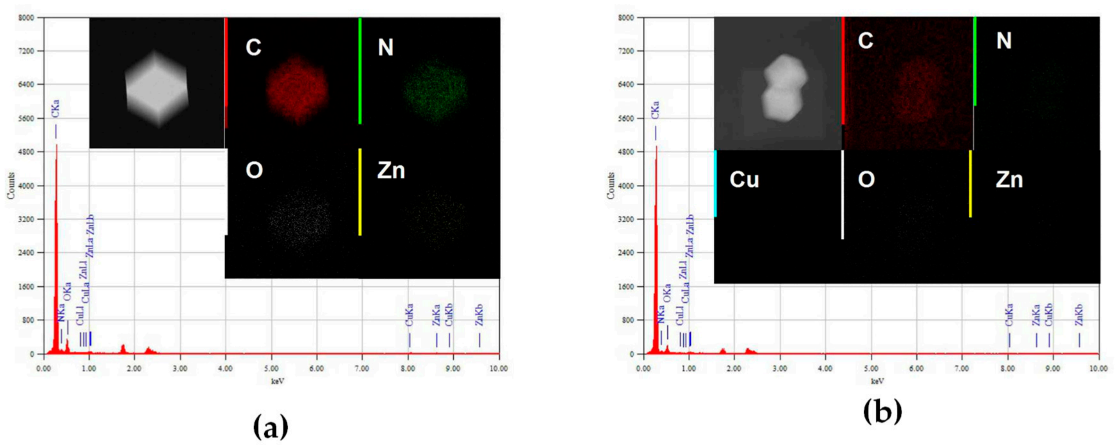

Figure S1. Elemental mapping images of (a) N-C and (b) Cu-N-C.

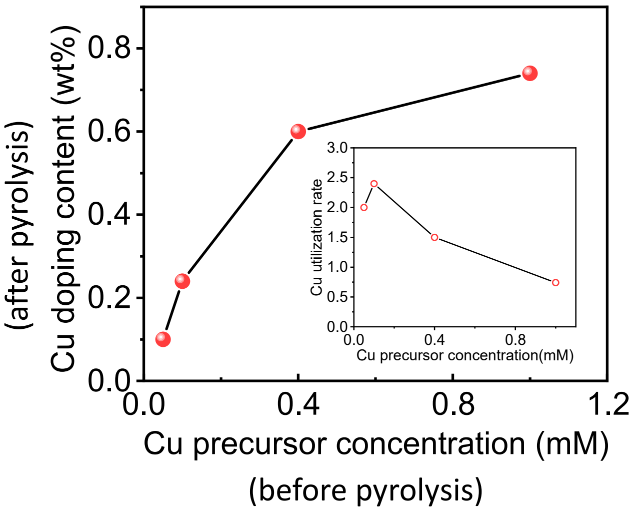

Figure S2. The Cu doping content and ratio of the catalysts prepared with different transition metal precursor concentrations. Routine condition: [catalyst] = 100 mg/L, [BPA] = 30 mg/L, [PMS] = 300 mg/L, temperature = 25°C, and initial pH = 7.0, reaction time = 11 min.

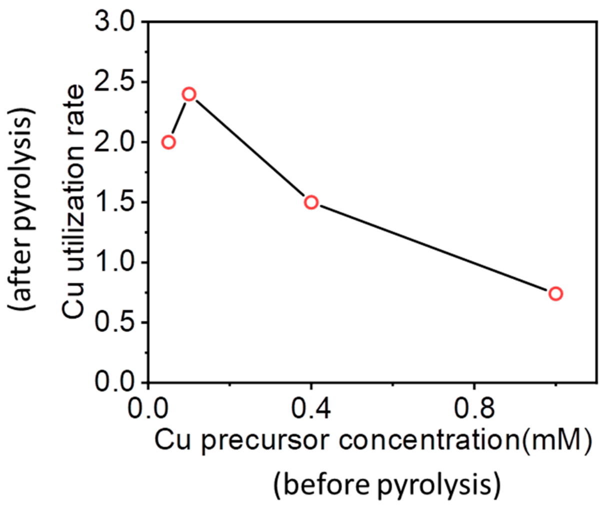

Figure S3. Metal leaching of Cu-N-C catalysts prepared by precursors in different concentration. Routine condition: [catalyst] = 100 mg/L, [BPA] = 30 mg/L, [PMS] = 300 mg/L, temperature = 25°C, and initial pH = 7.0, reaction time = 11 min.

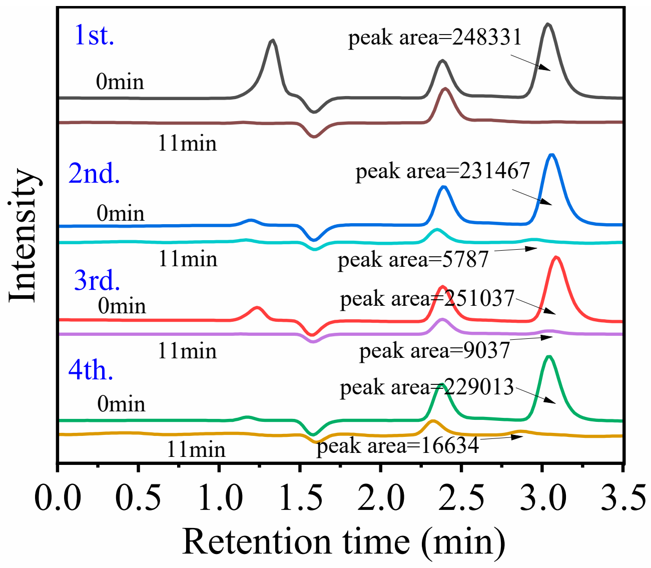

Figure S4. The chromatogram spectra of BPA in the four cycling experiments.

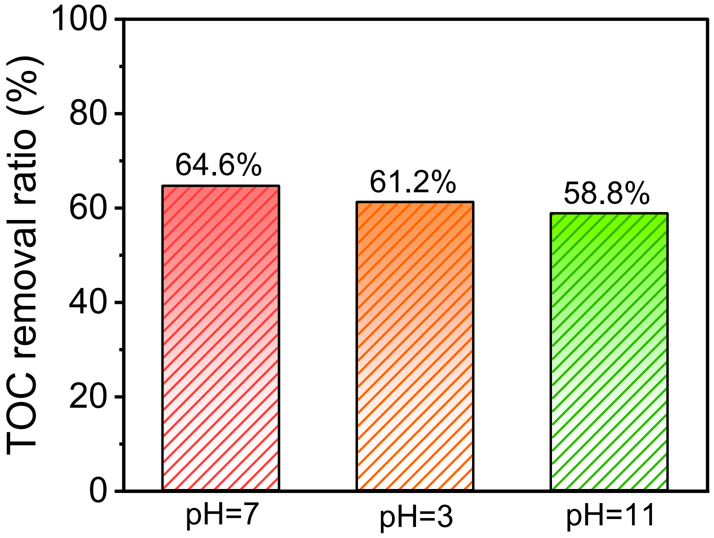

Figure S5. The mineralization of the BPA in Cu-N-C/PMS system under different pH values. Routine condition: [catalyst] = 100 mg/L, [contaminant] = 50 mg/L, [PMS] = 300 mg/L, temperature = 25°C, reaction time = 60 min.

Table S1. The comparison of catalytic performance for polymeric catalysts in PMS activation.

photocatalyst	catalyst loading (g/L)	PMS (mM)	pollutant	Target Conc. (mg/L)	η(t)%(min)	Ref
Cu-N-C	0.1	0.49	BPA	30	~99 (11)	This work
5%$NiCo_2O_4$/g-C_3N_4	0.5	1	CBZ	10	~98.5 (40)	[1]
COF-PRD	0.3	0.81	BPA	10	~100 (150)	[2]
5-ScO-CN	0.5	0.25	NB	25	~100 (120)	[3]
Fe^{3+}/g-C_3N_4	0.5	0.1	BPA	2.28	~100 (90)	[4]
g-C_3N_4	1.0	2	BPA	20	~30 (60)	[5]
PI-g-C_3N_4	1.0	5	BPA	10	~96 (60)	[6]
ACN450	0.25	1	DCP	7.7	~92 (90)	[7]
(1wt%) T/CN	0.25	0.4	DCF	10	~100 (40)	[8]

Reference

1. Peng, X.; Wu, J.; Zhao, Z.; Wang, X.; Dai, H.; Wei, Y.; Xu, G.; Hu, F., Activation of peroxymonosulfate by single atom Co-N-C catalysts for high-efficient removal of chloroquine phosphate via non-radical pathways: Electron-transfer mechanism. *Chemical Engineering Journal* **2022,** *429*.
2. Liu, F.; Dong, Q.; Nie, C.; Li, Z.; Zhang, B.; Han, P.; Yang, W.; Tong, M., Peroxymonosulfate enhanced photocatalytic degradation of serial bisphenols by metal-free covalent organic frameworks under visible light irradiation: mechanisms, degradation pathway and DFT calculation. *Chemical Engineering Journal* **2022,** *430*.
3. Sun, M.; Zhou, P.; Peng, J.; He, C.; Du, Y.; Pan, Z.; Su, S.; Dong, F.; Liu, Y.; Lai, B., Insights into peroxymonosulfate activation under visible Light: Sc2O3@C3N4 mediated photoexcited electron transfer. *Chemical Engineering Journal* **2022,** *435*.
4. Xu, L.; Qi, L.; Han, Y.; Lu, W.; Han, J.; Qiao, W.; Mei, X.; Pan, Y.; Song, K.; Ling, C.; Gan, L., Improvement of Fe2+/peroxymonosulfate oxidation of organic pollutants by promoting Fe2+ regeneration with visible light driven g-C3N4 photocatalysis. *Chemical Engineering Journal* **2022,** *430*.
5. Gong, Y.; Zhao, X.; Zhang, H.; Yang, B.; Xiao, K.; Guo, T.; Zhang, J.; Shao, H.; Wang, Y.; Yu, G., MOF-derived nitrogen doped carbon modified g-C3N4 heterostructure composite with enhanced photocatalytic activity for bisphenol A degradation with peroxymonosulfate under visible light irradiation. *Applied Catalysis B: Environmental* **2018,** *233*, 35-45.
6. Wang, G.; Zhao, Y.; Ma, H.; Zhang, C.; Dong, X.; Zhang, X., Enhanced peroxymonosulfate activation on dual active sites of N vacancy modified g-C(3)N(4) under visible-light assistance and its selective removal of organic pollutants. *Sci Total Environ* **2021,** *756*, 144139.
7. Cui, M.; Cui, K.; Liu, X.; Chen, X.; Chen, Y.; Guo, Z., Roles of alkali metal dopants and surface defects on polymeric carbon nitride in photocatalytic peroxymonosulfate activation towards water decontamination. *J Hazard Mater* **2022,**

424 (Pt A), 127292.

8. He, J.; Yang, J.; Jiang, F.; Liu, P.; Zhu, M., Photo-assisted peroxymonosulfate activation via 2D/2D heterostructure of Ti(3)C(2)/g-C(3)N(4) for degradation of diclofenac. *Chemosphere* **2020,** *258*, 127339.
